# AI-assisted radiographic identification of original vs. replica dental implants: comparing accuracy of human experts vs. probabilistic and deterministic AI

**DOI:** 10.1186/s40729-025-00662-2

**Published:** 2025-12-13

**Authors:** Mark K. Bremer, Maximilian Blume, Samir Abou-Ayash, Muhammad Naseer Bajwa, Sheraz Ahmed, Jochen Hardt, Katja Petrowski, Monika Bjelopavlovic

**Affiliations:** 1 Department of Prosthodontics and Material Science, University Medical Center Mainz, Augustusplatz 2, 55131 Mainz, Germany; 2 Department of Oral and Maxillofacial Surgery, University Medical Center Mainz, Augustusplatz 2, 55131 Mainz, Germany; 3 National University of Sciences and Technology, H-12, Islamabad, 44000 Pakistan; 4 National Centre of Artificial Intelligence, H-12, Islamabad, 44000 Pakistan; 5 German Research Center for Artificial Intelligence GmbH, 67663 Kaiserslautern, Germany; 6 Department of Psychosomatic Medicine and Psychotherapy, University Medical Center Mainz, Duesbergweg 6, 55128 Mainz, Germany

**Keywords:** Dental implant, Artificial intelligence, Deep learning, Diagnostic imaging, Detection algorithms

## Abstract

**Purpose:**

In dental implantology, the application of artificial intelligence (AI) for the differentiation of various implant systems is gaining increasing importance. This study investigates the feasibility of distinguishing between two highly similar implant (original implant and its replica) systems using an automated, AI-based recognition software.

**Methods:**

A dataset of 906 radiographic images was initially compiled, consisting of standardized ex situ recordings of both the original and the replica implants (with and without a cover screw in situ). Four deterministic AI-models and one probabilistic model were trained using different subsets of varying sizes of the dataset, including the full dataset and then evaluated against a designated test dataset. For comparison, 28 dental professionals also assessed the same test dataset.

**Results:**

The accuracy of the deterministic model trained solely with 488 radiographs of implants with inserted cover screws was 0.579 (57.9%). The second and third models, trained with a greater number of radiographs without inserted cover screws, achieved accuracies exceeding 0.90 and, in some instances, even reached 1.00. The fourth deterministic model, as well as the probabilistic model, comprising 28 classifiers and trained on the complete dataset, classified the test dataset without error. The dental professionals achieved an overall accuracy of 0.8616 (86.16%) in their assessment of the test dataset.

**Conclusion:**

This study suggests that AI-supported implant recognition software has the potential to offer valuable assistance in clinical practice for distinguishing between original and replica implants. Such differentiation can play a crucial role for prosthetic suprastructures and associated manufacturer warranties.

**Graphical abstract:**

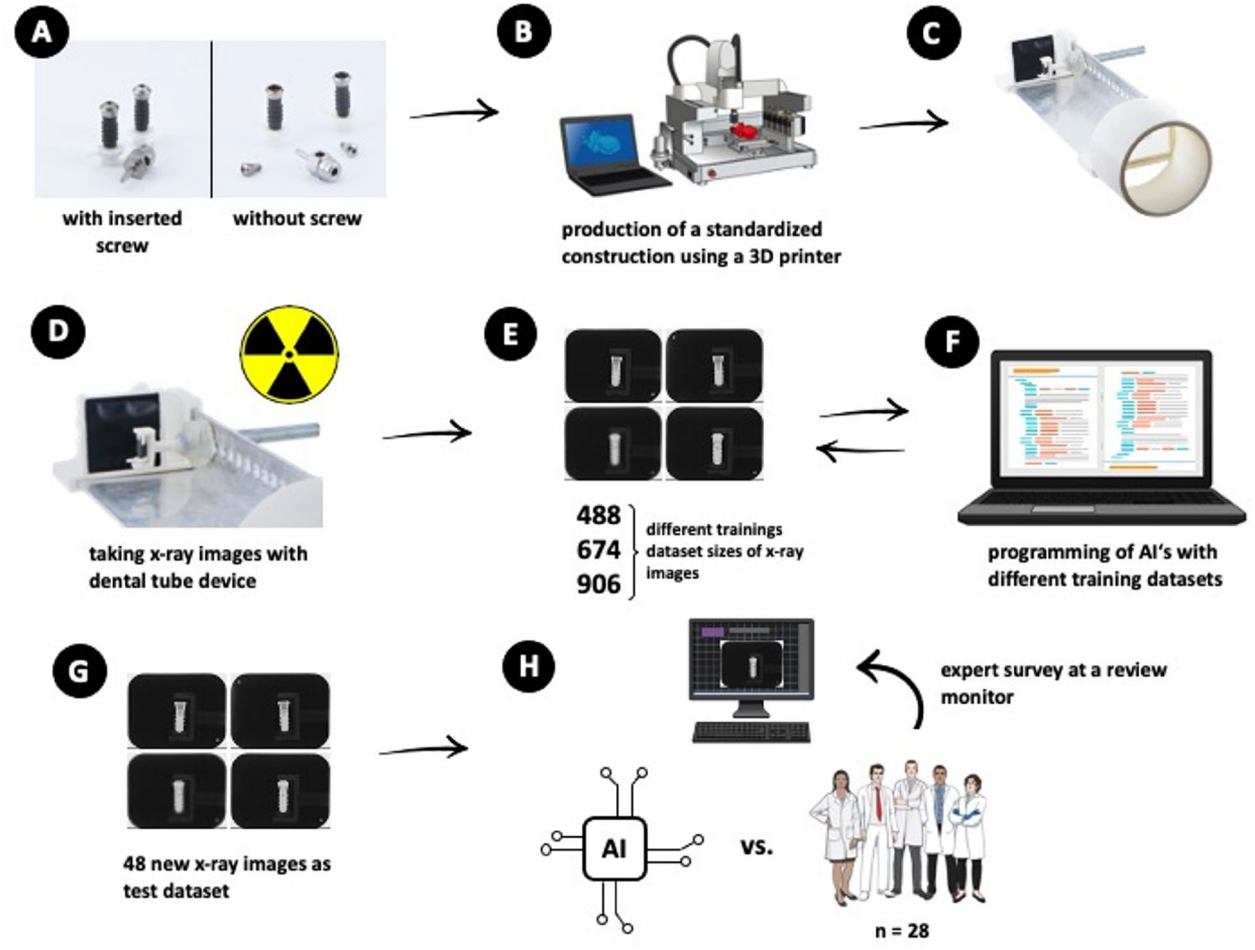

## Background

The rapid development of Artificial Intelligence (AI) and machine learning has led to significant advancements in dentistry over recent years [1], particularly in the field of automated image analysis. Artificial Neural Networks (ANNs), which are modeled on the principles of human brain information processing [2], are increasingly employed in image processing to deliver precise diagnoses and classifications of various diseases [3, 4]. In the dental context, special emphasis has been placed on the capabilities of deep learning-based models to identify dental implants from radiographic images. This process involves training a network, such as a Convolutional Neural Network (CNN), to analyse image data, detect patterns and features, and derive accurate classifications [5].

The dental implant market features a wide range of manufacturers, each offering distinct and proprietary designs. This diversity has resulted in a multitude of manufacturer-specific internal connections that are critical for the prosthetic suprastructure. Consequently, it is imperative for every treating clinician to have knowledge of the implant system in use. However, there is an increasing demand for restoring externally placed implants where the manufacturer is unknown, such as in cases of practice transitions with missing implant documentation, or implants placed abroad without sufficient records. This lack of information can lead to an inability to restore the implant or the fabrication of incorrect mesostructures. In such situations, clinicians often attempt to identify implant manufacturers based on characteristic structures observed in radiographic images. This suggests that AI and deep learning could be utilised to automate the identification of various implant systems from radiographs [6].

A previously overlooked area of research lies in the differentiation between original and replica implants on radiographs using AI. Replicas can be produced by other companies once a patent has expired. Studies have demonstrated that AI-models are capable of distinguishing implants based on subtle image features that are often difficult for the human eye to detect [7–11]. Dentists frequently face the challenge of reliably identifying the manufacturer of an implant on radiographs. However, it is crucial to establish absolute certainty in such cases to ensure the safety and stability of the prosthetic restoration [12–14]. This is essential for preserving patient health and achieving the necessary mechanical bond between the implant and its corresponding superstructure. A software solution capable of distinguishing between original and replica implants from digital radiographs would therefore prove highly valuable.

The present study aims to test the hypothesis that AI can differentiate between original and replica implants with greater accuracy than human experts. This hypothesis is based on previous findings regarding the efficiency of AI-driven image processing systems, which have demonstrated high classification accuracies in several studies [1, 3].

In recent years, an increasing number of studies have focused on the topic of automated implant identification, utilizing progressively larger and more diverse datasets. The heterogeneity of datasets used for training AI-models complicates comparisons of results and objective performance assessment.

This heterogeneity is reflected in the significant variability of reported results across studies. Accuracy rates range from a minimum of 67% [15] to a maximum of 98.51% [11]. Several studies in this field report accuracy values exceeding 90% [7–11, 16–22]. Systematic reviews have reported mean overall accuracy rates of 92.16% [3] and 92.56% [3] for the studies included.

## Methods

### Study design

The aim of this study was to classify two highly similar dental implants from different manufacturers using datasets segmented from extraoral radiographs of these implants. The datasets were initially gathered for training the AI-model with the manufacturers’ specifications used as reference standards to guarantee correct labeling of the data. These in vitro experiments were evaluated using several CNNs, including ResNet (Residual Neural Network). In a subsequent step, human experts assessed the same test dataset that was used to evaluate the accuracy of the AI-models. The results obtained from the AI-models and human experts were then compared. Statistical analyses, including chi-square tests, were conducted to compare outcomes between the original and the replica implant group. The overall study design is illustrated in the Graphical Abstract.

### Implants

**Fig. 1:**
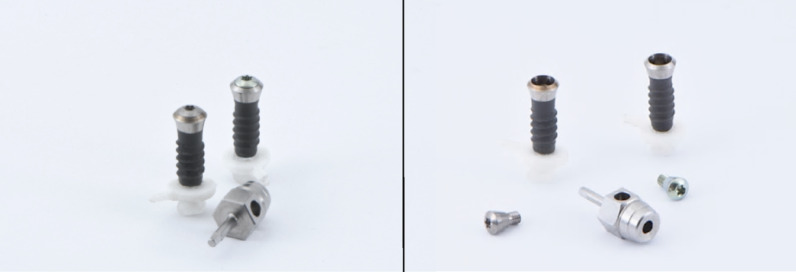
Original implant (left in each pair) and replica implant (right in each pair) with corresponding manual screwdriver; left: with inserted screw, right: without inserted screw

The implants used as the basis for comparison in this study are products from Straumann® (Basel/Switzerland) and Meisinger® (Neuss/Germany). The Straumann® implant (Standard Plus) represents the original product, while the corresponding replica is manufactured by Meisinger® (Fig. 1). Both implants share identical specifications in terms of shape, size, and diameter. The implant models each feature an endosseous length of 10 mm. The implant shoulder measures 1.8 mm in length in both cases, terminating at the soft tissue level. The endosseous diameter is 4.1 mm, while the implant platform diameter is 4.8 mm, measured at the widest point of the implant shoulder.

Due to the previously advertised compatibility of the replica implant and its components with the original product, clinicians were assured that components such as cover screws, impression posts, laboratory analogs and abutments from either manufacturer could be used interchangeably and safely without risk.

### Data acquisition

The dataset used to train the AI for distinguishing between the two described implants consisted of 906 radiographic images. These images were acquired ex situ, without prior clinical use of the implants. Until the initiation of the first image acquisition, all implants remained in their original packaging. A standardized imaging protocol was implemented to ensure consistent image quality across the dataset. The implants were positioned at various distances, inclinations and rotations relative to the detector, using a custom-designed fixation system tailored for this experimental setup.

**Fig. 2:**
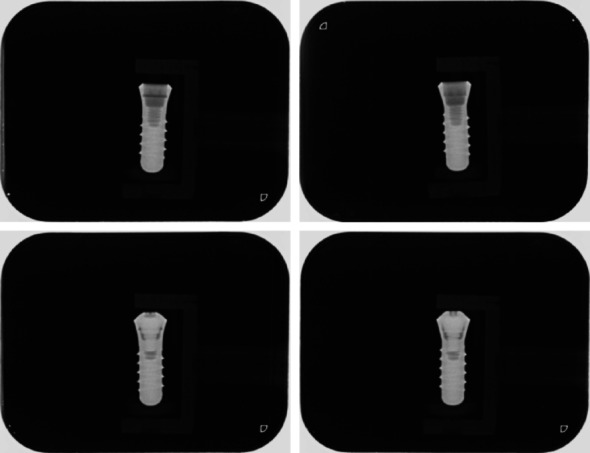
Representative radiographs of the implants included in the dataset; Top left: Straumann® implant without screw; Bottom left: Straumann® implant with screw; Top right: Meisinger® implant without screw; Bottom right: Meisinger® implant with screw

The distances ranged from 1 to 15 cm from the implant to the detector, resulting in variations in image size. Implant inclinations ranged from 0° to 45° in 5° increments, referring to the angle of the implant shoulder relative to the detector. Additionally, for each combination of distance and inclination, the implants were rotated around their longitudinal axis by 45°, 90° and 135°. Figure 2 displays four representative radiographs from the dataset.

The radiographs were captured using a dental X-ray tube device from Soredex (MinRay® model) with a focal spot size of 0.7 mm and an output of 60/70 kV at 7 mA. The images were recorded on phosphor storage plates (Dürr VistaScan Plus®, size 2, measuring 3 × 4 cm). After exposure, the plates were digitized using a Dürr VistaScan Mini View® scanner and imported into the Sidexis imaging software (version XG 2.65).

### Experimental setup

**Fig. 3:**
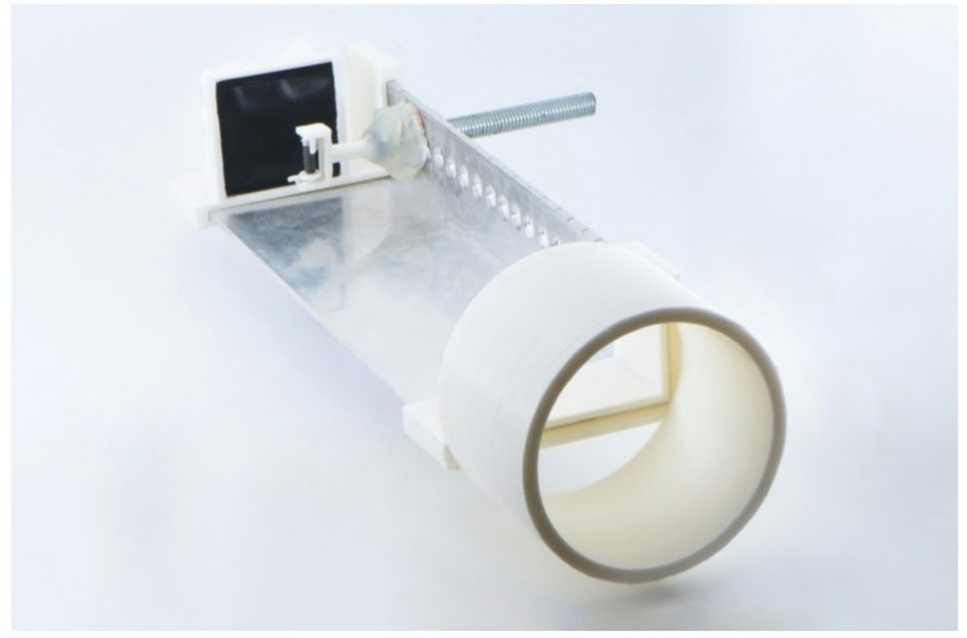
Complete construction for standardized imaging

The standardized imaging procedure was based on a custom-designed apparatus that allowed the implants to be fixed in the various described imaging positions while maintaining consistent geometry. This ensured that imaging was performed under standardized and reproducible conditions. The device was primarily fabricated using a 3D printer (i3 Mega S, Anycubic®) and supplemented with a stainless steel angle profile featuring evenly distributed drilled holes (Fig. 3).

### Training of the AI-model

This study experimented with multiple CNN architectures such as residual networks, inception networks, dense networks and hybrid architectures like inception-resnet models for training the AI-model for the detection of original and replica dental implants. Eventually ResNet-50-V2 was selected for its superior performance. All the models were trained following two training paradigms, namely deterministic and probabilistic. In a deterministic neural network, the weights of the network are estimated as a single scalar value for each parameter. Once these weights are learned, the model behaves deterministically, meaning it consistently produces identical results for the same input, as the network parameters are fixed constants. In contrast, a probabilistic neural network learns a probability distribution for each network parameter. After the training and during testing, a single value for each network parameter is sampled from their respective distributions and the resulting model is used to perform the inference. This approach enables drawing a posterior distribution on the output of the neural network. The variance of this posterior can then be used to estimate the uncertainty associated with the model’s prediction [23].

The models were incrementally trained on the data using k-fold cross-validation with k = 5. Initially, the training dataset consisted of 488 radiographs of the implant with the inserted screw containing 244 images per implant type (original and replica). Later the dataset was expanded with 93 images each for original and replica implants without containing any screws. Finally another 116 images of each implant type without a screw were added to make the total of 906 images.

To evaluate the accuracy of the different models, a completely independent test dataset was created. This test dataset consisted of 48 radiographs of the implants, including both with and without the screw images. The images were equally distributed across the four categories: originals with or without cover screw and replicas with or without cover screw. The imaging distances were 1.5 cm or 7.5 cm. Rotations were set to 67.5°, 90°, 112.5° or 135° and tilt angles to 2.5°, 7.5° or 12.5°. The AI-model and its developer had no access to the correct labeling of this data, ensuring that the evaluation of the index test was performed under blinded conditions.

### Study participants

The participant group consisted of individuals working in the field of dentistry, including dental students (n = 19) from their second to fifth clinical semester, as well as practicing dentists from a university clinic (n = 9). Their experience in dentistry ranged from a minimum of three and a half years to a maximum of nine years (mean = 5.29 years). A total of 28 dental professionals participated in the survey. Regarding gender distribution, the group comprised n = 15 female and n = 13 male participants.

### Study procedure

Before the survey, participants were presented with four analog sample images from the training dataset. Each image depicted the implant at a distance of 1 cm from the detector, with an inclination of 0° and a rotation of 45° around its longitudinal axis. These sample images represented one of the four image categories included in this study (Fig. 2). Participants were given four minutes to familiarize themselves with the characteristics of the implants.

The diagnostic display and assessment setup were situated in an environment corresponding to a dental imaging workstation, classified as Room Class 5. Prior to the survey, the room was darkened, reducing ambient light levels to below 100 lx to ensure optimal conditions for radiological image evaluation.

The participants were subsequently presented with the radiographs from the test dataset in a randomized order. During this phase, they were not allowed to reference the sample images for orientation. Each radiograph was displayed for a maximum duration of one minute. Participants could manually proceed to the next image by clicking a mouse but were unable to revisit previous images. A Fujitsu display monitor (model B22W-6) with a screen resolution of 1680 × 1050 (WSXGA +) was used for image presentation.

**Fig. 4:**
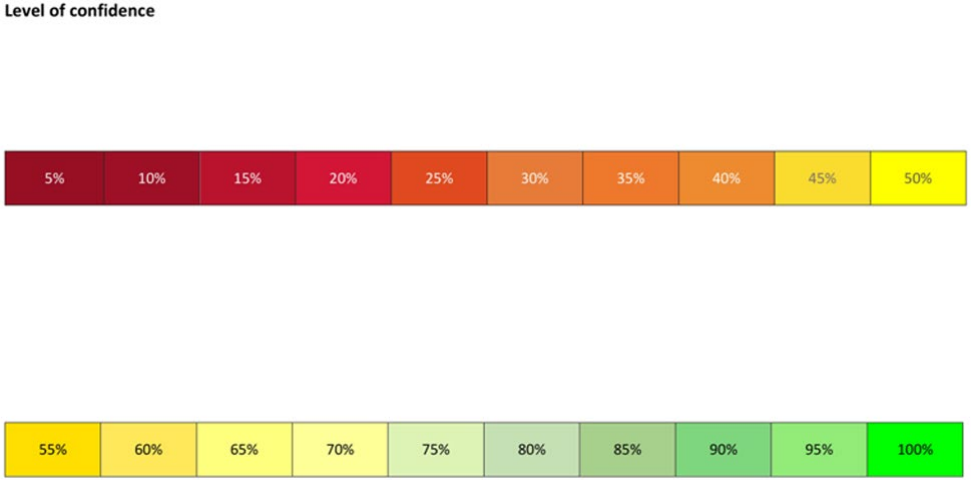
Visual reference for categorizing participant confidence levels

For each radiograph, participants were required to determine whether it depicted an original or a replica implant. Additionally they were asked to report their confidence level to enable comparison with the results of the AI-model. A visual scale ranging from 0 to 100%, divided into 5% increments, was used to indicate the participant’s confidence in their decision. A confidence level of 0% reflected no certainty regarding the correctness of their decision, whereas a confidence level of 100% indicated absolute certainty in the accuracy of their choice (Fig. 4). No radiographs were excluded, and no missing data occurred. All acquired images fulfilled the predefined quality criteria and were included in the analysis. Diagnostic performance metrics (accuracy and chi-square statistics) were calculated in accordance with STARD recommendations.

## Results

### Results of the convolutional neural network

The accuracy of the deterministic fivefold cross-validation model, trained exclusively on 488 radiographs of implants with an inserted screw, achieved an overall accuracy of 57.9% on the test dataset. Specifically, it identified radiographs of the original implant with an accuracy of 54.2% and the replica implant with an accuracy of 61.7% (Table 1).

**Table 1:** (False classifier = 5): Training dataset = 488 images; images with screw = 488, images without screw = 0; *Χ*^2^: Chi-squared value, df: degrees of freedom, p: p-value

	% correct	*Χ*^2^	df	p
Total	57.92			
Original	54.17			
Replica	61.67	6.05	1	.014

**Fig. 5:**
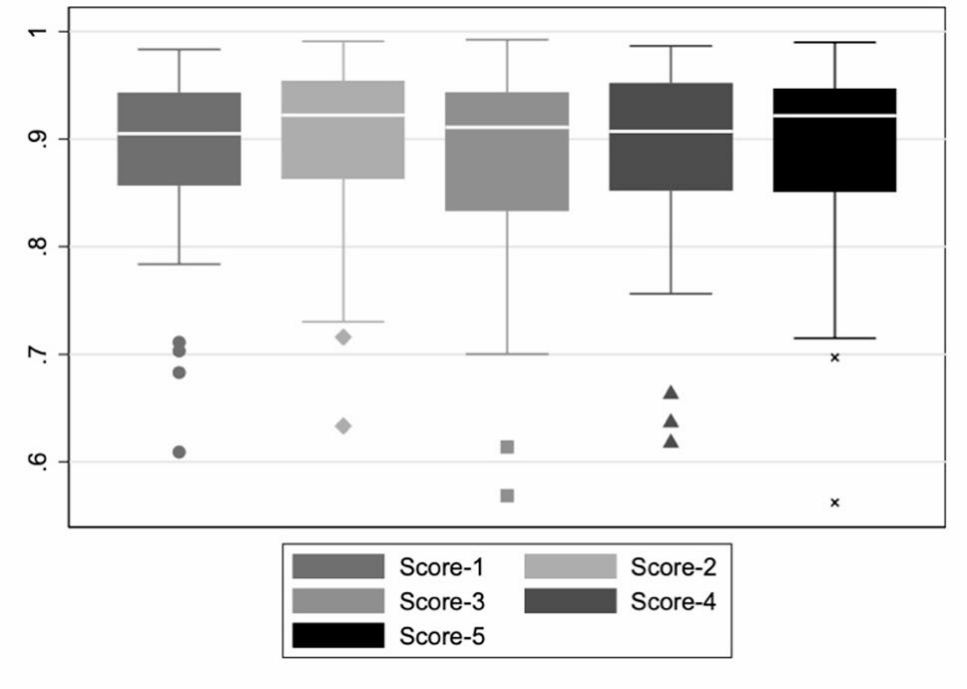
Training dataset = 674 images; images with screw = 488, images without screw = 186

**Fig. 6:**
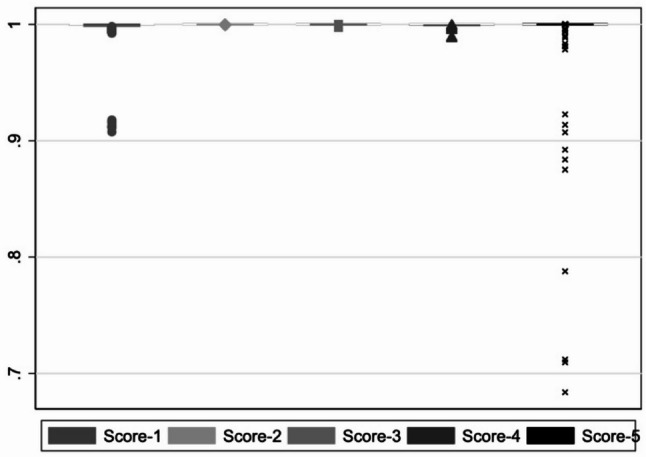
Training dataset = 906 images; images with screw = 488, images without screw = 418

The second deterministic model, which included a dataset of 674 images, achieved a classification accuracy of > 90% across all five classifiers for the test dataset (Fig. 5). The five scores shown in Figs. 5 and 6 represent the outputs of the different classifiers used to evaluate the test datasets for each AI-model. Each classifier assigns a pseudoprobability (score) for a given input, indicating the model’s confidence that the input belongs to a specific class.

In contrast, the accuracy of the third model was significantly improved by more than doubling the number of radiographs without an inserted screw to a total of 418. As a result, the second and third classifiers achieved an accuracy of 100% (Fig. 6).

**Fig. 7:**
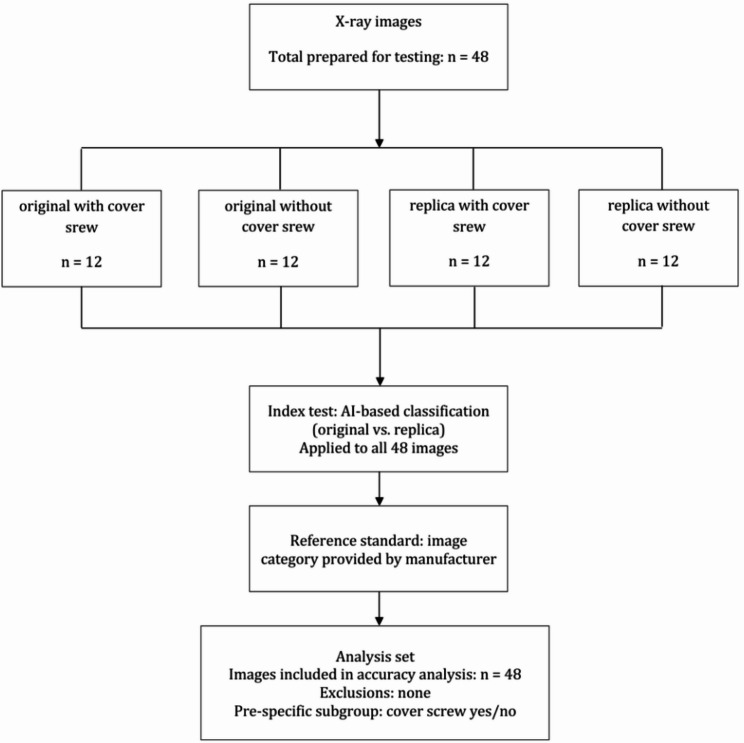
Flow diagram of participants (participants = X-ray images)

The deterministic and probabilistic models, each utilizing 28 classifiers, achieved an accuracy of 100% across all classifiers (Fig. 7).

### Results of the study participants

The study participants demonstrated an overall accuracy of 86.16% for the test dataset. The original implant was identified with a higher success rate, achieving an accuracy of 88.69%, whereas the replica implant was correctly identified with an accuracy of 83.63%. The individual results are summarized in Table 2.

**Table 2:** Accuracy rates of human experts; *Χ*^2^: Chi-squared value, df: degrees of freedom, p: p-value

	% correct	*Χ*^2^	df	p
Total	86.16			
Original	88.69			
Replica	83.63	705	1	< .001

There were differences in performance between female and male participants. Female participants achieved an overall accuracy of 84.86%, with approximately 8% higher accuracy in identifying the original implant compared to the replica implant (Table 3). Male participants achieved an overall accuracy of 87.66%, with a 1.6% higher accuracy in identifying the original implant compared to the replica implant (Table 4).

**Table 3:** Accuracy rates of female experts; *Χ*^2^: Chi-squared value, df: degrees of freedom, p: p-value

	% correct	*Χ*^2^	df	p
Total	84.86			
Original	88.89			
Replica	80.83	352	1	< .001

**Table 4:** Accuracy rates of male experts; *Χ*^2^: Chi-squared value, df: degrees of freedom, p: p-value

	% correct	*Χ*^2^	df	p
Total	87.66			
Original	88.46			
Replica	86.86	354	1	< .001

**Fig. 8:**
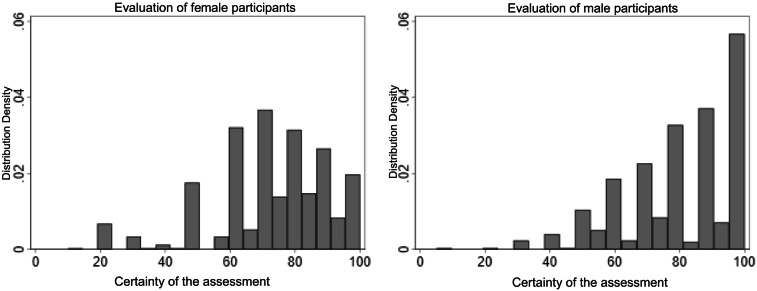
Confidence levels of male and female study participants

In addition, the confidence levels reported by the groups varied. The distribution of confidence levels among female participants showed a wide range across the scale (Fig. 8), indicating that many women expressed moderate to high confidence in their judgments. However, relatively few female participants reported maximum confidence (100%) (n = 6).

In contrast, the confidence levels among male participants were more concentrated at higher values. Notably, there was a significant density at the maximum confidence level of 100%, suggesting that several male participants made their judgments with the highest level of certainty.

## Discussion

The objective of this study was to evaluate the performance of AI in distinguishing between original and replica dental implants in radiographs, comparing it with the accuracy of human experts. Our findings reveal that the performance of AI in implant classification is heavily influenced by the size and diversity of the training dataset, supporting the hypothesis that AI can outperform human experts when adequately trained on large, diverse datasets [24].

Contrary to our initial expectation, AI trained on a smaller dataset of 488 images, which excluded those without an inserted screw, demonstrated an overall accuracy lower than that of human experts. Specifically, the accuracy of the AI-model was 57.9%, significantly lower than the 86.16% accuracy achieved by the study participants. This outcome highlights a critical limitation in the use of small and homogeneous datasets for training AI-models. AI-models trained on a limited set of images may struggle to generalize, especially when they encounter variations in the dataset that are not well-represented during training [25].

However, when the dataset was expanded to include a greater number of images, particularly with the addition of 674 and 906 radiographs containing variations such as the presence or absence of an inserted screw, the AI’s performance improved substantially. The models trained on these larger, more comprehensive datasets demonstrated accuracy levels exceeding 90%, with the highest accuracy reaching 100% in some classifiers. These results corroborate the idea that AI-models benefit from exposure to a diverse range of input data, which allows the algorithms to learn more complex and varied features that may be crucial for accurate implant classification. This underscores the importance of data augmentation in developing AI systems that can effectively handle the variability encountered in clinical settings [26].

In comparison, the human experts in our study showed an overall accuracy of 86.16%, with a slightly better success rate in identifying the original implant (88.69%) versus the replica implant (83.63%). Although human performance was consistent across the participants, with little variation due to gender, the confidence levels reported by participants suggested a range of certainty in their diagnoses, with some participants expressing high levels of confidence while others displayed more uncertainty. This suggests that factors such as experience and familiarity with implant systems may influence human decision-making, which may be addressed with further training or clinical exposure. Previous studies have shown that decision-making processes vary with clinical experience, which may influence diagnostic confidence and accuracy [27].

While AI-models demonstrated superior accuracy when trained on larger datasets, human experts remained competitive, particularly in scenarios where implant identification was more straightforward. It is worth noting that AI, even with extensive datasets, may still face challenges in distinguishing highly similar implants due to the subtle nature of distinguishing features that may not be easily visible in radiographic images. Moreover, the reliance on the visual perception of human experts may account for their relatively higher accuracy in situations where the implant differences are apparent, albeit subtle. Studies could already demonstrate the possibilities to distinguish between different implant systems especially for clinical decision-making [24].

Additionally, while the AI systems in this study showed strong results with sufficient data, it is crucial to assess their real-world applicability. In clinical practice, AI systems must be able to operate within the context of the variability inherent in patient anatomy, implant placement and image quality. Implementing AI-driven tools in everyday clinical workflows will require further validation across different populations, imaging devices and clinical conditions to ensure reliable and accurate implant identification.

### Limitations and future research

This study has several limitations. First, the dataset used for training the AI was restricted to a single implant system pair (one original and its corresponding replica). As a result, the findings cannot be readily generalized to other implant platforms or replica designs. Second, all radiographs were obtained under a highly standardized in-vitro setup using fixed imaging parameters. Such controlled conditions do not fully reflect the variability of clinical radiographs, which are influenced by patient anatomy, implant angulation, exposure differences, and operator-dependent factors. Consequently, the real-world performance of the models may differ from the results observed in this study. Additionally, the final test dataset consisted of only 48 radiographs (12 per category). This limited sample size may not fully represent the variability encountered in real clinical imaging and could contribute to an overestimation of model performance, particularly for models that achieved 100% accuracy. Larger and more heterogeneous test sets are therefore required for more robust validation.

While the accuracy of the deterministic and probabilistic models improved markedly with larger datasets, distinguishing original from replica implants remains a challenging task due to the subtle nature of radiographic differences. In addition, more advanced AI architectures and uncertainty-aware approaches should be explored using clinical recall radiographs to better capture the diversity and complexity of routine practice.

Future research should involve multi-manufacturer datasets including various implant lines, larger numbers of original–replica pairs, and real clinical radiographs obtained under heterogeneous imaging conditions. Such studies are essential to validate the robustness, clinical transferability and generalizability of both deterministic and probabilistic AI-models.

## Conclusions

The study demonstrates that AI-based systems, particularly when trained on larger and standardised datasets, are capable of achieving high accuracy in distinguishing between two highly similar implants [28]. Nonetheless, it remains essential to acknowledge that human expertise continues to play a key role [29], especially in clinical contexts where the judgement of medical professionals is indispensable but demonstrates less exact results than those of AI [17, 30]. In implant dentistry, replica implants demonstrate a possible problem in the daily practice, especially when no implant data is available and suprastructure components are used from the original implant system. While the results demonstrate technical feasibility under laboratory conditions, further validation using clinical radiographs is essential before any routine clinical application can be recommended.

## Data Availability

The data from this study were part of the dissertation paper from M.K.B. Data can be seen in Figs. 2, 5, 6 and 8; Tables 1, 2, 3 and 4. The datasets used and/or analysed during the current study are available from the corresponding author on reasonable request.

## Abbreviations

AI: Artificial Intelligence
ANN: Artificial Neural Network
CNN: Convolutional Neural Network
ResNet: Residual Neural Network
